# Antidiabetic Effects of Anthocyanins on Pancreatic β-Cell Function: A Systematic Review of *In Vitro* Studies

**DOI:** 10.3390/ijms27031415

**Published:** 2026-01-30

**Authors:** Ravish Kumkum, Theresha Ruwan Pathiranage, Bryony A. McNeill, Leni R. Rivera, Kathryn Aston-Mourney

**Affiliations:** IMPACT, Institute for Mental and Physical Health and Clinical Translation, School of Medicine, Deakin University, Geelong 3220, Australia; k.ravish@deakin.edu.au (R.K.); s223308588@deakin.edu.au (T.R.P.); bryony.mcneill@deakin.edu.au (B.A.M.)

**Keywords:** anthocyanin, type 2 diabetes, pancreatic β-cell, insulin secretion, antidiabetic properties, cyanidin-3-glucoside

## Abstract

Pancreatic β-cell dysfunction is the key driver of type 2 diabetes, and anthocyanins have been proposed as dietary compounds that may help preserve β-cell health. This systematic review aimed to synthesise evidence on the direct effects of anthocyanins on β-cell viability, apoptosis, oxidative stress, and insulin secretion across *in vitro* models. Four databases were searched in March–April 2025, and eighteen studies met the inclusion criteria. Purified anthocyanins—including cyanidin-3-glucoside (C3G), cyanidin-3-rutinoside (C3R), malvidin-3-glucoside (M3G), and delphinidin-3-glucoside (D3G)—as well as anthocyanin-rich berry extracts, were tested in INS-1, MIN6, RIN-m5F cells and primary mouse or human islets under glucotoxic, lipotoxic, oxidative, cytokine, and amyloidogenic stress. Anthocyanins consistently improved β-cell viability, reduced apoptosis, and lowered reactive oxygen species (ROS), nitric oxide (NO), and thiobarbituric acid reactive substances (TBARSs) levels while enhancing antioxidant enzyme activities. Multiple studies showed upregulation of insulin secretion-related genes and proteins, and both acute and chronic treatments increased glucose-stimulated insulin secretion under normal and stressed conditions. Mechanistic pathways involved modulation of mitogen-activated protein kinase (MAPK) signalling, endoplasmic reticulum (ER) stress responses, inflammatory mediators, and mitophagy (PINK1/PARKIN). While effective *in vitro* concentrations were higher than typical circulating levels, the collective evidence highlights anthocyanins as promising β-cell protective agents and underscores the need for studies examining their metabolites and physiologically relevant exposure.

## 1. Introduction

Diabetes has emerged as one of the fastest-growing global health challenges of the 21st century, affecting an estimated 589 million adults in 2024, with projections reaching 853 million by 2050 [1]. The disease is responsible for over 3.4 million deaths annually and accounts for more than one trillion USD in global healthcare expenditure [1]. Beyond these direct impacts, diabetes substantially increases risks of cardiovascular disease [2], kidney failure [3], cancers [4,5], and a wide range of infectious diseases [6,7]. Type 2 diabetes (T2D) constitutes the vast majority of global diabetes cases and is primarily driven by two physiological impairments: (i) insulin resistance and (ii) progressive pancreatic β-cell dysfunction [8]. While insulin resistance is a key pathophysiological component of T2D, β-cell dysfunction, encompassing impaired function and loss of mass, represents the critical determinant of disease progression and onset of hyperglycaemia [8]. In addition, β-cell damage is often irreversible, making restoration of lost mass or function challenging, whereas insulin resistance often improves with lifestyle or pharmacological interventions [9,10,11]. This underscores the importance of β-cell preservation as a therapeutic priority.

β-cells operate under substantial physiological demand, and in states of overnutrition and obesity, they face persistent exposure to elevated glucose and circulating lipids [8,12]. This chronic metabolic stress initiates glucotoxic and lipotoxic cascades, including oxidative stress, endoplasmic reticulum (ER) stress, mitochondrial impairment, and inflammatory signalling [13,14]. Compounding this vulnerability is the inherently low expression of antioxidant enzymes in pancreatic islets, which limits their capacity to neutralise reactive oxygen species (ROS); as a result, oxidative stress plays a central role in driving β-cell dysfunction during the development of diabetes [15]. Together, these insults compromise insulin biosynthesis, blunt glucose-stimulated insulin secretion, and impair cellular survival.

Human β-cells are particularly vulnerable to these stressors. Unlike rodent β-cells, which retain some regenerative capacity, adult human β-cells exhibit minimal proliferation or renewal [16]. Consequently, T2D progression is driven largely by the cumulative loss of existing functional β-cell mass rather than insufficient regeneration [17]. This decline often involves dedifferentiation, whereby stressed β-cells lose their mature identity and downregulate key transcription factors such as pancreatic and duodenal homeobox 1 (PDX*-1*) and MAF BZIP Transcription Factor A (MAFA) [18]. As these cells progressively lose their ability to respond to glucose, compensatory mechanisms fail, accelerating hyperglycaemia and disease progression [19].

A range of pharmacological agents, including metformin, insulin secretagogues, incretin-based therapies, and sodium–glucose co-transporter 2 (SGLT2) inhibitors, are currently used to manage T2D [20]. While these medications improve glycaemic control and alleviate some metabolic burden on β-cells, their protective effects are incomplete and often transient [21]. They do not fully prevent long-term β-cell decline, may lose efficacy as the disease progresses, and are associated with adherence-limiting side effects, such as gastrointestinal intolerance, hypoglycaemia risk (with insulin secretagogues), weight changes, genitourinary infections, and high cost [21,22]. Crucially, current drugs do not directly address the underlying cellular insults, particularly oxidative and ER stress, that drive β-cell dysfunction and loss. These limitations highlight a growing need for complementary strategies that are safe, well-tolerated, and capable of reducing the underlying cellular stressors that drive β-cell dysfunction.

In this context, dietary approaches have attracted increasing interest due to their accessibility and favourable safety profiles. Among dietary polyphenols, anthocyanins, a subclass of flavonoids responsible for the red, purple, and blue pigmentation of many fruits and vegetables, have gained considerable attention for their potential to modulate β-cell function [7]. These compounds are abundant in berries, grapes, black rice, purple corn, and other pigmented plants, and have been shown to enhance insulin secretion, improve cell viability, reduce apoptosis, and protect against glucolipotoxicity [23,24]. Their effects are mediated in part through regulation of key β-cell genes, such as PDX-1 (a core β-cell identity transcription factor), glucose transporters involved in β-cell glucose sensing (e.g., GLUT2 in rodents; GLUT1/GLUT3 in humans), and GPR40 (a fatty-acid-responsive receptor that amplifies insulin secretion), as well as through mitigation of oxidative stress [25,26]. Preclinical studies further suggest that anthocyanins contribute to improved glucose homeostasis by enhancing insulin sensitivity, modulating glucose metabolism, and preserving β-cell integrity under metabolic stress [7]. Specific anthocyanins, including glycosides of cyanidin, delphinidin, pelargonidin, and malvidin, have demonstrated protective effects by reducing oxidative damage and regulating molecular pathways involved in insulin secretion and cell survival [27,28,29,30,31]. To elucidate these mechanisms, numerous *in vitro* studies using isolated islets and β-cell lines have explored these cytoprotective and insulinotropic effects [32,33,34]; however, outcomes vary with anthocyanin type, concentration, cell model, and experimental design. Furthermore, while dietary anthocyanins are extensively metabolised in vivo, most *in vitro* studies have necessarily focused on parent compounds, leaving the direct effects of their physiological metabolites on β-cell function largely unexplored. As a result, despite growing evidence, current understanding remains fragmented, and a comprehensive systematic evaluation of anthocyanin effects on β-cells in the context of diabetes is still lacking.

Therefore, this systematic review aims to synthesise and critically evaluate existing evidence on the effects of anthocyanins and anthocyanin-rich extracts (AREs) on β-cell function, focusing on outcomes, including cell viability, apoptosis, oxidative stress, and glucose-stimulated insulin secretion (GSIS). By focusing on *in vitro* models that allow examination of β-cell responses under controlled conditions, this review aims to clarify key mechanisms and assess the potential of anthocyanins to directly protect β-cell function.

## 2. Methods

### 2.1. Search Strategy

**Figure 1:**
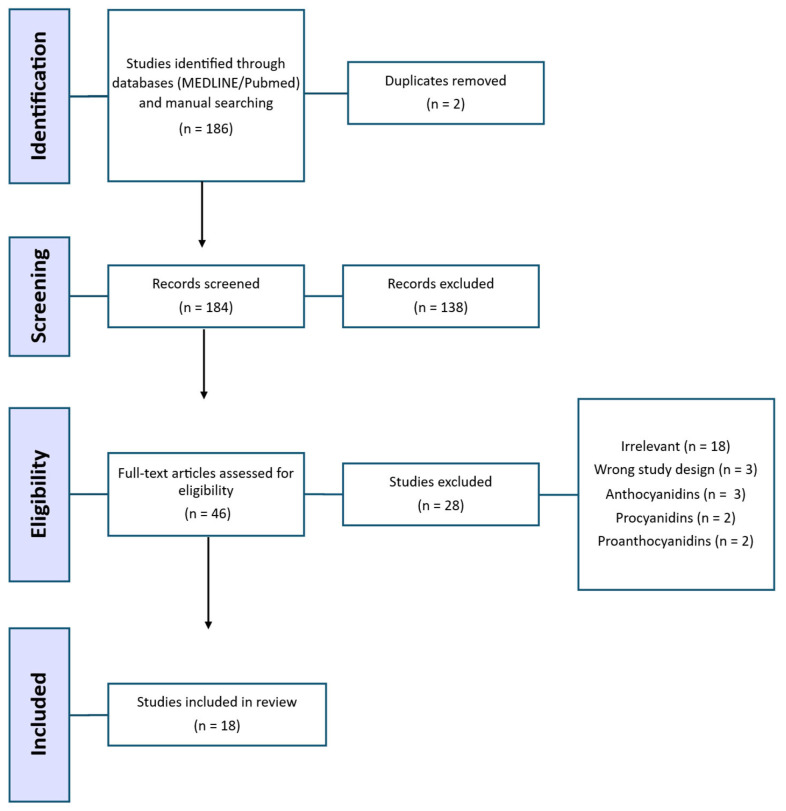
PRISMA flow diagram of study selection for the systematic review.

This systematic review was conducted in accordance with the Preferred Reporting Items for Systematic Reviews and Meta-Analyses (PRISMA) guidelines (Figure 1) [17]. The review was preregistered on the Open Science Framework on 13 November 2025 (https://doi.org/10.17605/OSF.IO/EJPKY). A comprehensive literature search was performed in PubMed, Web of Science, Scopus, and the Virtual Health Library (VHL) between March and April 2025 to identify studies examining the effects of anthocyanins on pancreatic β-cells in the context of diabetes. Search terms, combined keywords, and MeSH terms related to:

(a) anthocyanins (e.g., glycosylated forms of cyanidin, delphinidin, pelargonidin, peonidin, petunidin, malvidin); (b) pancreatic β-cells (e.g., “islet cells,” “pancreatic islets,” “insulin-producing cells”); and (c) diabetes-related terms (e.g., type 1 diabetes, type 2 diabetes, T1DM, T2DM, hyperglycemia/hyperglycaemia insulin resistance).

The final search string was:

((anthocyanin* OR cyanidin* OR delphinidin* OR pelargonidin* OR peonidin* OR petunidin* OR malvidin*) AND (“beta cell*” OR “pancreatic beta cell*” OR “islet cell*” OR “pancreatic islet*” OR “insulin-producing cell*”) AND (diabetes OR diabetic OR “Type 1 diabetes” OR “Type 2 diabetes” OR T1DM OR T2DM OR hyperglycemia OR hyperglycaemia “insulin resistance”))

OR ((“Anthocyanins” [MeSH] OR “Flavonoids” [MeSH]) AND (“Islets of Langerhans”[MeSH] OR “Insulin-Secreting Cells”[MeSH]) AND (“Diabetes Mellitus”[MeSH] OR Diabetes)). In PubMed, the MEDLINE filter was applied, retrieving 167 articles before screening.

### 2.2. Inclusion and Exclusion Criteria

Studies were included if they investigated glycosylated anthocyanins or anthocyanin-rich extracts using *in vitro* β-cell or islet models and reported at least one outcome related to β-cell function, insulin secretion, viability, apoptosis, oxidative stress, or gene expression related to these pathways (Table 1). Only original research articles published in English up to April 2025 were considered. Studies were excluded if they used in vivo models, ex vivo tissue or organ preparations, or clinical models; focused on compounds other than anthocyanins; lacked relevant outcome measures; or were reviews, editorials, or conference abstracts.

### 2.3. Compound Classification and Exclusion Rationale

For this review, only glycosylated anthocyanins were included, as these represent the predominant dietary and physiologically relevant forms found in fruits and vegetables [35,36]. Anthocyanidins, the aglycone counterparts, were excluded due to their limited dietary presence, lower stability, and distinct bioavailability and metabolic profiles, which could confound interpretation of anthocyanin-specific effects on β-cell function. This restriction ensured methodological consistency and dietary relevance across included studies.

### 2.4. Study Selection Process

All stages of the review process, title/abstract screening, full-text review, and final inclusion, were conducted using Covidence, Veritas Health Innovation, Melbourne, Australia (https://www.covidence.org, accessed on 20 April 2025). Two independent reviewers screened the articles. Disagreements were resolved by consensus.

### 2.5. Data Extraction

Quantitative and qualitative data were independently extracted by two reviewers into a structured Excel spreadsheet. Extracted information included publication details (title, year, journal), study design (β-cell or islet type, experimental insult, and exposure duration), and anthocyanin characteristics (type, concentration, and source). Relevant outcome measures encompassed insulin secretion, cell viability, apoptosis, antioxidant activity, gene expression, and associated signalling pathways. When data were presented graphically, values were extracted using WebPlotDigitizer version 5 (https://automeris.io/WebPlotDigitizer/, accessed on 15 June 2025).

### 2.6. Quality Assessment

The methodological quality of all included *in vitro* studies was evaluated using a modified ToxRTool framework (https://joint-research-centre.ec.europa.eu/scientific-tools-and-databases-0/toxrtool-toxicological-data-reliability-assessment-tool_en, accessed on 1 August 2025) [37]. Twelve criteria distributed across five domains, compound characterisation, experimental model details, study design, data transparency, and mechanistic insight, were assessed. While the original ToxRTool applies a binary (0–1) scoring system, criteria in the present review were adapted to a 0–2 scale to distinguish between absent (0), partially reported (1), and adequately reported (2) methodological information, a common challenge in reporting of *in vitro* studies. Total scores (maximum = 24) were used to categorise studies as high (≥16), medium (12–15), or low (<12) quality, ensuring standardised evaluation of study reliability and reporting completeness.

### 2.7. Data Synthesis and Assessment of Heterogeneity

Heterogeneity was assessed qualitatively by comparing experimental designs, biological models (e.g., cell lines vs. primary islets), interventions (purified anthocyanins vs. extracts), stress models (e.g., glucotoxicity, lipotoxicity, oxidative stress), and differences in outcome normalisation and reporting (e.g., insulin secretion expressed per protein content, per cell number, or in culture supernatants); as a result, quantitative synthesis by meta-analysis was deemed inappropriate. Consequently, a narrative synthesis was employed. Studies were grouped thematically based on reported outcomes (e.g., GSIS, viability, apoptosis, gene expression), and quantitative trends were summarised descriptively using means, standard errors, and sample sizes where available.

## 3. Results

### 3.1. Study Characteristics

A total of 186 records were identified through database searches (MEDLINE/PubMed) and manual searching, including reference lists, citation tracking, and grey literature sources. Following screening, 18 studies met the inclusion criteria and were included in the systematic review, with their key characteristics summarised in Table 2.

The 18 included studies investigated the effects of anthocyanins on β-cells using an *in vitro* experimental design. Two main categories of β-cell models were used: primary islets and β-cell lines. Primary islets were derived from mouse or human pancreas, while β-cell lines were derived from rat or mouse, including INS-1, INS-1 832/13, and RIN-m5F (rat) and MIN6, MIN6N, and NIT (mouse). Among the 18 included studies, two utilized islets, two employed both islets and β-cell lines, and the remaining 14 used rat or mouse β-cell lines only. Anthocyanins were tested either as purified compounds, such as cyanidin-3-glucoside (C3G), cyanidin-3-rutinoside (C3R), malvidin-3-glucoside (M3G), delphinidin-3-glucoside (D3G), and cyanidin-3-galactoside (C3Gal), or as anthocyanin-rich extracts (AREs) from sources including Chinese bayberry, mulberry, aronia, blueberry, red maize, and Cornelian cherries. Treatment concentrations varied depending on the anthocyanin type and source. To ensure consistency and enable comparison across studies, all concentrations of purified anthocyanins were standardised to µM using available molecular weight information, while ARE were standardised to µg/mL. Reported concentrations for purified anthocyanins ranged from 0.1 to 500 µM, with exposure durations typically between 8 and 48 h. Nine studies evaluated anthocyanin effects on β-cells under stress conditions relevant to T2D pathophysiology, including glucotoxic stress (glucose, 25 to 30 mM), lipotoxic stress (palmitic acid, 0.2 to 0.5 mM), glucolipotoxic stress (combined high glucose and palmitic acid), oxidative stress (hydrogen peroxide), amyloidogenic stress (amylin and amyloid-β_1_–_42_ (Aβ_1_–_42_), 10 µM), and cytokine-induced inflammatory stress (IFN-γ and IL-1β). The outcomes assessed included cell viability (15 studies), apoptosis (7 studies), insulin secretion (12 studies), antioxidant activity (9 studies), and gene or protein expression relating to apoptosis, insulin secretion, and antioxidant activity (12 studies).

### 3.2. Quality Assessment

All studies were assessed for methodological quality using a modified ToxRTool, with 94% classified as high quality and 6% as medium quality; none were low quality (Table 3). Strengths (high scores) were prominent in several domains, including exposure conditions, statistical analysis, variability reporting, controls, source/preparation, and replicates. Limitations (low scores) primarily involved concentration rationale (criterion 3; mean 1.0), cell source details (mean 1.2, with 78% scoring ≤ 1), mechanistic insight (mean 1.4, 44% ≤ 1), and disclosure of limitations/conflicts (mean 0.9, 28% ≤ 0.5). Overall, the evidence demonstrates generally high methodological quality, tempered by gaps in concentration justification and reporting transparency.

### 3.3. Cell Viability and Cytotoxicity

A total of 14 studies assessed the impact of anthocyanins on β-cell viability using various assays. The most commonly used method was the MTT assay, followed by trypan blue exclusion, CCK-8, and Calcein-AM/EthD-1 Live/Dead staining.

Under non-stressed conditions, M3G [30] and berry ARE (1–1000 µg/mL) [40,45] showed no effects on β-cell viability at concentrations up to 100 µM for M3G, 10 µg/mL for blueberry ARE, and 1000 µg/mL for aronia ARE. Notably, purified anthocyanins C3G [38] and C3R [33] increased β-cell viability, with maximal effects observed at 1–5 µM and 100 µM, respectively. A cytotoxic effect was observed at higher concentrations, including 300 µM M3G [30], ≥445 µM C3G [32], and at 100 µg/mL blueberry ARE [45]. In human islets, 1 µM C3G showed no effect under non-stressed conditions [39].

Under glucotoxic conditions, both C3G [29] and C3R [27] increased β-cell viability in a dose-dependent manner compared to the stressed control. Similarly, under glucolipotoxic conditions, viability was improved in a dose-dependent manner by blueberry ARE (10–100 µg/mL) [45] but showed no effect under non-stressed conditions. Under lipotoxic conditions, C3G also enhanced β-cell viability, with maximal effects observed at 50 µM [42].

Under oxidative stress, anthocyanin treatment resulted in higher viability relative to stressed controls across INS-1, MIN6, mouse islet, and human islet models. Five studies reported cytoprotective effects using both purified anthocyanins and ARE derived from bayberry [28,39,44] and mulberry [32,43] with effective concentrations ranging from 0.1 to 50 µM for bayberry C3G to 12.5–668 µM for mulberry anthocyanins. Lastly, under cytokine-induced inflammatory stress (IL-1β + IFN-γ), aronia berry ARE (1–1000 µg/mL) similarly preserved β-cell viability, showing no reduction compared with non-stressed controls [40].

### 3.4. Apoptosis, Oxidative Stress, and Inflammation Pathways

Seven studies examined anthocyanin effects on β-cell apoptosis using Annexin V-FITC/PI dual-label staining and caspase-based assays, while oxidative stress markers and antioxidant enzyme activities were assessed concurrently in most of these studies.

Under chronic glucotoxic stress, mulberry-derived C3G (111–156 µM) reduced apoptosis in MIN6 cells in a dose-dependent manner [29]. The same concentrations decreased apoptosis in INS-1 cells while lowering intracellular ROS generation [29]. In another glucolipotoxic model, C3G (89–178 µM) enhanced antioxidant defences, increasing superoxide dismutase (SOD) and catalase (CAT) activities and elevating PINK1, PARKIN, and LC3 proteins, alongside reduced mitochondrial ROS [42]. C3R (50 µM) similarly decreased apoptosis in INS-1 cells, with substantial reductions in nitric oxide (NO), ROS, and thiobarbituric acid reactive substances (TBARSs) levels (45–55%) and concurrent increases in SOD, CAT, and glutathione peroxidase (GSH-Px) activities [27]. At the molecular level, C3R increased the *Bcl2/Bax* ratio and downregulated caspase-3, caspase-9, and cytochrome c protein expression [27].

Under lipotoxic conditions, commercially sourced C3G (50 µM) reduced apoptosis in INS-1 cells and primary mouse islets, accompanied by a modulation of GPR40 (fatty-acid receptor), toll-like receptor 4 (TLR4, an inflammatory receptor), ER-stress markers (PERK, CHOP, GRP78, eIF2α), inflammatory cytokines, including tumour necrosis factor-alpha (TNF-α) and interleukin-1 beta (IL-1β), apoptosis-related proteins (cleaved caspase, BCL-2, BAX), and the glucose transporter GLUT2 [26].

Similarly, under glucolipotoxic conditions, C3G (89–178 µM) enhanced superoxide dismutase (SOD) and catalase (CAT) activities, increased PINK1, PARKIN, and LC3 protein levels, and reduced ROS [42]. Furthermore, blueberry ARE (10–100 µg/mL) reduced apoptosis in INS-1 832/13 cells under glucolipotoxic stress, while also increasing antioxidant enzyme activities, including SOD and GSH-Px, and lowering ROS and malondialdehyde levels [45].

Under oxidative stress, anthocyanins consistently reduced apoptosis across multiple β-cell types. Chinese bayberry-derived C3G (1 µM) decreased apoptosis in INS-1 cells and mouse islets, accompanied by reduced ROS generation and upregulation of *Ho-1* mRNA expression [34], while bayberry ARE reduced ROS levels [28]. In MIN6 cells, mulberry-derived C3G and C3R (≤50 µM) reduced apoptosis while increasing glucokinase (GK), glucagon-like peptide-1 receptor (GLP-1R), and pancreatic and duodenal homeobox 1 (PDX-1) proteins [43]. Meanwhile, in MIN6N cells, C3G (~156–445 µM) increased ROS-scavenging and lipid-peroxidation inhibition activities and markedly inhibited H2O2-induced apoptotic cell death by 32% [32].

Under cytokine-induced stress, aronia berry ARE (1–1000 µg/mL) decreased NO production in RIN-m5F cells and downregulated key inflammatory signalling pathways, including cyclooxygenase-2 (COX-2), inducible nitric oxide synthase (iNOS), mitogen-activated protein kinases (MAPKs; ERK, JNK, p38), and nuclear factor kappa B (NF-κB) [40].

Under amyloidogenic stress in human islets, C3G (1 µM, 24 h) reduced ROS generation induced by amylin or amyloid-β_1_–_42_ (Aβ_1_–_42_), upregulated the antioxidant enzyme heme oxygenase-1 (HO-1) and the autophagy marker LC3, and downregulated NOD-, LRR- and pyrin domain-containing protein 3 (NLRP3) and IL-1β [39].

### 3.5. Insulin Secretion

Insulin secretion is a key indicator of β-cell function and is sensitive to metabolic and cellular stress. Twelve of the 18 studies included in this review examined the effects of anthocyanins on insulin secretion under diverse experimental conditions. These included non-stressed conditions, as well as pathological stress models relevant to T2D, such as high glucose (glucotoxicity), palmitic acid-induced lipotoxicity, oxidative stress, and amyloidogenic peptide exposure. Secretion was assessed using static incubations, with basal glucose (2–11 mM) used to measure baseline insulin secretion (BIS) and higher glucose (10–25 mM) used to assess GSIS. Studies were classified as acute (0.3–2 h exposure) or chronic (12–48 h exposure) to distinguish immediate regulatory effects from longer-term adaptations.

#### 3.5.1. Acute Effects on Insulin Secretion

For acute conditions, anthocyanins demonstrated a direct insulinotropic effect across multiple β-cell models. In the absence of stress, M3G (100 µM) [30] and C3R (60–300 µM) [33] significantly increased GSIS in INS-1 cells [30]. Similarly, another study examined the effects of four anthocyanins from Cornelian cherry—C3G, D3G, C3Gal, and P3G—on insulin secretion in INS-1 832/13 cells [31]. C3G and D3G were the most effective, increasing both BIS and GSIS, while P3G caused a modest increase in BIS. However, C3Gal showed only marginal effects under the tested conditions [31].

#### 3.5.2. Chronic Effects on Insulin Secretion

Under chronic stress conditions, anthocyanin treatment consistently preserved or restored secretory capacity across different models of metabolic dysfunction. Specifically, in models of glucotoxicity, anthocyanin pre-treatment enhanced insulin secretion. For example, C3G from mulberry (156 µM) increased GSIS in MIN6N cells [29], and C3R (50 µM) improved both BIS and GSIS in INS-1 cells [27]. This protective efficacy extended to lipid-induced stress. Under lipotoxic stress, C3G (50 µM) co-treated with palmitic acid increased GSIS in INS-1E cells and by a notably larger extent in primary mouse islets [26]. Similarly, Chinese blueberry ARE (1–100 µg/mL) ameliorated glucolipotoxicity-induced dysfunction in INS-1 832/13 cells by dose-dependently reducing elevated basal insulin release while simultaneously enhancing GSIS [45]. Under oxidative stress conditions, anthocyanins from mulberry and bayberry preserved β-cell insulin secretion. In MIN6N cells, C3G (~156 µM) increased BIS [32]. Similarly, in MIN6 cells, pretreatment with C3G or C3R (prior to H_2_O_2_ exposure) enhanced both basal and GSIS in a dose-dependent manner, with maximal effects at 50 µM [43]. Lastly, in human islets exposed to amyloidogenic stress, pretreatment with bayberry C3G (1 µM) followed by co-exposure to amylin or Aβ_1_–_42_ increased GSIS at 20 mM glucose by about 2.5-fold in amylin-treated islets and 1.2-fold in Aβ_1_–_42_ stressed islets [39].

#### 3.5.3. Molecular Mechanisms: Gene and Protein Expression

In addition to the changes in insulin secretion, several studies assessed concomitant changes in gene and protein expression to elucidate the mechanisms underlying these functional improvements.

At the transcriptional level, anthocyanin treatment consistently upregulated genes involved in glucose sensing and the secretory machinery. In INS-1 cells, purified anthocyanins (C3G, C3R, and M3G) upregulated genes critical for insulin synthesis and K_ATP_- channel-dependent secretion, including *Ins1*, *Ins2*, *Slc2a2* (GLUT2), *Gck*, *Cacna1c*, and *Kcnj11* (Kir6.2) [26,30,33]. Consistent with these findings, ARE from Chinese bayberry were also shown to significantly upregulate *Ins2* and *Pdx-1* [28].

At the protein and signalling level, anthocyanins upregulated the protein expression of key metabolic regulators and receptors. Red maize ARE increased the protein expression of phospholipase C (PLC), free fatty acid receptor 1 (FFAR1/GPR40), and phosphorylated protein kinase D (p-PKD) [41]. Likewise, blueberry/blackberry ARE enhanced GSIS concurrently with the upregulation of the GLP-1 receptor (GLP-1R) and IGF-1 receptor (IGF-1R), alongside more than 20 other genes related to insulin signalling [25]. Furthermore, C3G and C3R were shown to increase protein levels of Glucokinase (GK), PDX-1, and GLP-1R in MIN6 cells [43], while bayberry ARE increased total insulin protein content [28].

## 4. Discussion

This systematic review synthesises evidence from 18 *in vitro* studies demonstrating that anthocyanins exert multifaceted protective effects on β-cells under T2D-like conditions. Both purified anthocyanins and ARE consistently improved cell viability, reduced apoptosis, and preserved insulin secretory function across glucotoxic, lipotoxic, oxidative, and amyloidogenic stress models. These benefits were mediated through antioxidant, anti-inflammatory, and anti-apoptotic pathways, with dose-dependent responses observed across multiple β-cell models.

Most studies utilised β-cell lines, with only four studies employing primary islets. Primary islets are heterogeneous, containing multiple endocrine cell types, and better mimic in vivo conditions through paracrine signalling and physiological insulin secretion in response to glucose [46]. In contrast, β-cell lines provide a homogeneous and reproducible system but lack the complex interactions of whole islets [47]. It should be noted that these differences could affect both the magnitude and mechanisms of anthocyanin effects. Similarly, anthocyanins were administered either as purified compounds or ARE, which also contain other polyphenols. The results from these two forms were considered separately, as the effects of extracts likely reflect synergistic interactions between anthocyanins and co-occurring polyphenols.

Anthocyanins protected β-cell viability under various stress conditions, with effects most pronounced under glucotoxic, oxidative, or glucolipotoxic stress. Purified C3G increased viability at concentrations ranging from 0.5 µM to 156 µM [44], while M3G and C3R showed no effects at lower concentrations, with cytotoxicity at higher concentrations (≥100–300 µM). ARE from various sources, including blueberry, blackberry, bayberry, and red maize, also enhanced cell viability under stress, whereas aronia berry ARE showed no effect both at basal and under cytokine stress. All studies except one assessed viability using the MTT assay, which measures mitochondrial metabolic activity rather than true cell survival [48]. Consequently, changes in mitochondrial function or redox state can influence readings independent of actual cell survival. Given anthocyanins’ antioxidant and mitochondrial-modulating properties, MTT results may overestimate viability, as reported for other polyphenol-rich treatments [49]. Additionally, MTT outcomes can be strongly affected by cell type, incubation conditions, and assay duration [49,50]. Importantly, seven studies complemented viability measurements with apoptosis assays (Annexin V/PI staining, caspase activity), and the concordance between reduced apoptosis and improved viability supports genuine cytoprotection beyond mitochondrial effects. Therefore, future studies should employ multiple complementary approaches to assess β-cell viability and death, such as trypan blue exclusion, ATP-based luminescence assays, live/dead staining, and apoptosis markers (e.g., Annexin V/PI or caspase activity), alongside MTT, to validate cytoprotective effects and avoid overestimation of viability [51,52].

β-cell apoptosis is a major contributor to the decline in insulin secretion observed throughout the progression of diabetes [53]. Across seven studies, anthocyanins from bayberry, mulberry, and blueberry consistently reduced apoptosis under oxidative, glucolipotoxic, and high-glucose stress, with effective concentrations ranging from 1 µM to 70 µM in both β-cell lines and primary islets. These anti-apoptotic effects paralleled improvements in cell viability, supporting the interpretation that anthocyanins preserve β-cell survival by attenuating programmed cell death pathways. Further, apoptosis in β-cells is primarily regulated via the intrinsic (mitochondrial) and extrinsic pathways, with the intrinsic pathway involving B-cell lymphoma 2 (Bcl-2) family proteins, mitochondrial membrane potential, and cytochrome c release leading to caspase activation, while the extrinsic pathway involves death receptor signalling and caspase-8 activation [53]. Stress-responsive MAPK signalling pathways (ERK, JNK, p38) further modulate these apoptotic responses to cellular insults [53].

Under glucotoxic or oxidative stress, β-cells typically exhibit increased Bax expression, cytochrome c release, caspase activation, and NF-κB nuclear translocation [27,29]. In contrast, H_2_O_2_-induced oxidative stress generates excessive ROS that disrupts mitochondrial membrane potential, similarly activating MAPK signalling and the intrinsic apoptotic pathway [32]. Several studies indicate that C3G and C3R attenuate these responses through suppression of MAPK phosphorylation, restoration of the Bcl-2/Bax ratio, reduction of cytochrome c-mediated caspase activation, and prevention of NF-κB translocation [29,32]. Additionally, C3G enhances β-cell survival and function associated with upregulation of PDX-1, GK, and GLP-1 receptor levels, while reducing ROS production [32]. These mechanistic insights are further supported by *in vitro* and in silico studies showing that other anthocyanins, including D3G, Pel3G, and P3G, may exert anti-apoptotic effects in β-cells against glucolipotoxicity by the same mechanism [54,55]. At the molecular level, anthocyanins consistently upregulated anti-apoptotic factors, including Bcl-2 and Survivin, which inhibit apoptotic pathways and maintain functional β-cell mass, while downregulating pro-apoptotic factors, including Caspase-3, Caspase-9, and Cytochrome c [27,38].

Beyond classical apoptotic regulation, anthocyanins also activated several complementary stress-response pathways. C3G upregulated HO-1, an oxidative stress-responsive enzyme, through PI3K/Akt and ERK1/2 pathways, and modulated markers of the unfolded protein response (PERK, CHOP, GRP78), thereby protecting β-cells from dysfunction caused by misfolded proteins [26,38,39]. Anthocyanins further suppressed inflammatory mediators, including COX-2, iNOS, IL-1β, and NLRP3, which reduced cytokine-induced damage [26,40]. Additionally, upregulation of autophagy-related markers, including LC3 and activation of the PINK1/PARKIN mitophagy pathway, were observed in some studies, indicating a potential role for anthocyanins in promoting mitochondrial turnover, reducing ROS accumulation, and supporting the energy demands of insulin secretion [39,42]. These findings are broadly consistent with the anti-apoptotic and insulin-enhancing actions reported for other polyphenols, such as resveratrol, quercetin, and genistein in β-cells, which appear to act through similar pathways [56].

β-cells are particularly susceptible to oxidative stress due to their low intrinsic antioxidant capacity [57]. Excessive ROS production, stemming from mitochondrial respiration or metabolic stress, can damage DNA, mitochondria, and cell membranes, thereby impairing β-cell function and survival [57]. Seven of the studies included in this review reported that anthocyanins, including C3G, C3R, and ARE from mulberry, aronia, and blueberry, mitigated oxidative damage by reducing ROS, NO, and TBARS levels, while enhancing the activities of endogenous antioxidant enzymes such as SOD, CAT, and GSH-Px [27,29,32,39,40,42,45]. Evidence from these studies suggests that C3G alleviates oxidative stress in β-cells in part by promoting mitophagy via the PINK1/PARKIN pathway, facilitating the removal of damaged mitochondria and limiting further ROS generation (23,35). This effect, together with the upregulation of SOD, CAT, and GSH-Px, contributes to the restoration of redox homeostasis in hyperglycaemic and glucolipotoxic β-cells [40]. Similarly, C3R was reported to enhance antioxidant enzyme activity, including SOD, CAT, and GSH-Px, in β-cells exposed to high-glucose conditions, supporting the notion of a conserved antioxidant mechanism [27]. These findings indicate that anthocyanins preserve β-cell viability and function not only by reducing oxidative damage but also by supporting mitochondrial quality and endogenous antioxidant defences, complementing their insulinotropic effects.

Progressive deterioration of insulin secretory capacity represents a hallmark of T2D pathophysiology. Twelve of the included studies demonstrated that anthocyanins—including C3G, C3R, M3G, D3G, P3G, and C3Gal—and ARE significantly increased GSIS across β-cell lines and primary islets under both basal and stressed conditions. Effective concentrations ranged from 1 µM in human islets to 50–70 µM in rodent cells, with dose-dependent effects particularly pronounced under glucotoxic, glucolipotoxic, oxidative, and amyloidogenic stress. Importantly, these insulinotropic effects occurred concurrently with enhanced viability and reduced apoptosis, indicating that anthocyanins preserve both functional capacity and cell survival. Crucially, several studies noted no adverse effects on viability or insulin secretion in non-stressed control cells. This selectivity is clinically favourable, suggesting that anthocyanins do not disrupt the homeostasis of healthy, well-functioning β-cells, but specifically exert a protective and restorative effect when cells are compromised by metabolic stress.

**Figure 2:**
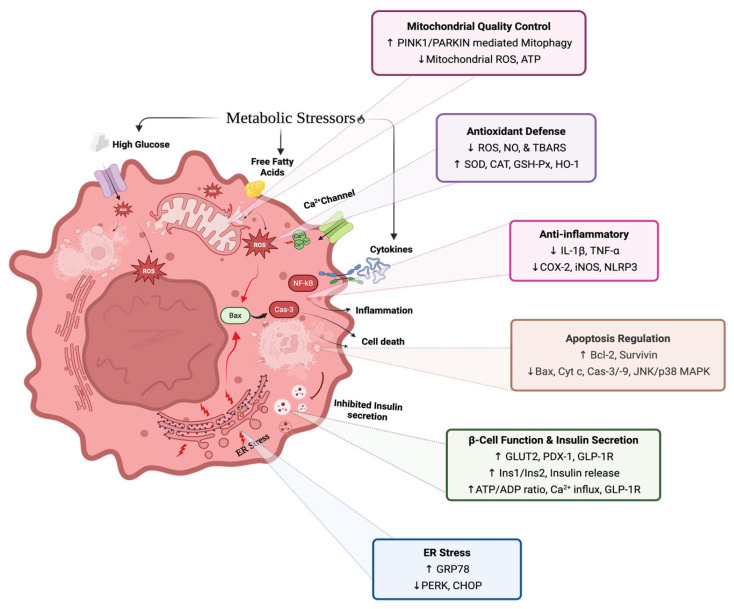
Anthocyanin-modulated protective mechanisms in pancreatic β-cells under metabolic stress. Metabolic stressors, including high glucose, free fatty acids, and cytokines, induce oxidative stress, inflammation, endoplasmic reticulum (ER) stress, mitochondrial dysfunction, apoptosis, and impaired insulin secretion in β-cells. Anthocyanins attenuate these stress-induced damages by enhancing antioxidant defence, suppressing inflammatory signalling, regulating apoptotic pathways, improving mitochondrial quality control, alleviating ER stress, and preserving β-cell function and insulin secretion (Created in https://BioRender.com, accessed on 23 January 2026). Upward (↑) and downward (↓) arrows indicate increased and decreased levels or activity, respectively.

A limited number of studies included in this review directly investigated the mechanistic pathways underlying the insulinotropic effects of anthocyanins. Findings from these studies suggest that improvements in insulin secretion may involve coordinated influences on glucose sensing, Ca^2+^ signalling, and insulin synthesis pathways [33]. C3G emerged across several studies as a broadly protective anthocyanin, improving β-cell function under both glucotoxic and oxidative stress by easing ER stress, reducing apoptosis, and supporting insulin synthesis and secretion. In comparison, C3R was reported to enhance insulin secretion without affecting cell viability, acting through intracellular Ca^2+^ signalling and increased ATP production via GK upregulation [33]. At the transcriptional level, several anthocyanins upregulated genes central to β-cell glucose sensing and insulin production, including *Ins1/Ins2*, GLUT2 (Slc2a2), Gck, and the transcription factor PDX-1, while also modulating ion channel genes such as Cav1.2 and Kir6.2 that influence membrane excitability and exocytosis [26,28,30,33,39]. Upregulation of GLP-1R further enhances β-cell responsiveness to incretin hormones, potentiating insulin secretion in a glucose-dependent manner [25,26]. These findings align with broader evidence demonstrating that dietary polyphenols protect β-cells from metabolic stress. Similar protective effects have been reported for several flavonoids, including quercetin and epigallocatechin gallate, which also modulate oxidative stress, ER stress, and apoptotic pathways in β-cells [58]. Overall, these mechanisms highlight that anthocyanins act through multiple interconnected pathways relevant to β-cell function, consistent with broader evidence that many natural products exert anti-diabetic effects via multi-target and network-based mechanisms rather than single molecular targets as shown in preclinical models [59,60]. A schematic summary of the protective mechanisms of anthocyanins in pancreatic β-cells is presented in Figure 2.

As anthocyanins are known to rapidly metabolise, they typically reach only nanomolar plasma concentrations after dietary intake (1.4–200 nM < 2 h) [61]. For example, Matsumoto et al. reported peak plasma levels of 5–73 nM after blackcurrant anthocyanin ingestion [62], while another human pharmacokinetic study found total anthocyanin concentrations of 55–168 nM following a high 720 mg dose, with most compounds eliminated within 4 h [63]. In contrast, studies included in this review reported beneficial β-cell effects following treatment concentrations of 1–100 µM, doses which far exceed typical circulating concentrations. Comparable beneficial effects may still be physiologically relevant in vivo through tissue accumulation, chronic low-dose exposure, or bioactive metabolites. Importantly, although anthocyanins are rapidly absorbed and extensively metabolised following dietary intake, their phase II and gut microbiota-derived phenolic metabolites, such as protocatechuic, vanillic, and ferulic acids, circulate at substantially higher and more sustained concentrations than parent compounds [64,65,66]. These metabolites exhibit anti-inflammatory [67] and antioxidative [68] activities comparable to, or even exceeding, those of parent anthocyanins. Notably, specific metabolites such as protocatechuic acid [69] and vanillic acid [70] have demonstrated anti-diabetic properties when administered as individual compounds, including improvements in glycaemic control and metabolic stress markers. Consistent with this, both C3G and protocatechuic acid have been shown to exert insulin-like effects via activation of PPARγ in human adipocytes [67], supporting the biological relevance of anthocyanin metabolites in modulating glucose homeostasis. However, this fundamental difference between *in vitro* conditions and typical in vivo circulating concentrations highlights the need for studies examining anthocyanin metabolites and chronic low-dose exposure using pre-clinical models.

Supporting their translational relevance, epidemiological evidence shows inverse associations between anthocyanin intake and T2D risk [71], while intervention studies show that anthocyanin-rich berries improve glycaemic control and insulin sensitivity in individuals with insulin resistance [72,73]. The present review provides mechanistic insight into these clinical observations, suggesting that β-cell protection may contribute to the anti-diabetic effects of anthocyanin-rich foods alongside their known effects on insulin sensitivity and inflammation.

Several methodological and translational limitations should be taken into account. Firstly, most of the studies examined relied on immortalised β-cell lines and short exposure periods, potentially limiting their direct physiological relevance, and only two used human islets. Considerable heterogeneity in anthocyanin sources, purity, and extraction methods complicates comparison, and therefore, the results from pure ACN and ARE should be interpreted with caution. Further, the acute high-dose stressors commonly used (25–30 mM glucose, 0.5 mM palmitate) only partially model the chronic, lower-grade metabolic stress of T2D. Future work should prioritise longer-term exposure models, assessment of physiologically relevant metabolites, and greater use of human islets or stem cell-derived β-cells. Mechanistic depth could be strengthened through single-cell omics, while validation in islet perfusion systems and in vivo diabetic models remains essential to translate these findings. Studies exploring anthocyanin combinations with other dietary polyphenols may also help define effective nutritional strategies.

Overall, this systematic review highlights the robust potential of anthocyanins to preserve β-cell function and survival under metabolic stress conditions characteristic of T2D. By targeting distinct apoptotic and oxidative pathways specifically within compromised cells, anthocyanins offer a promising therapeutic avenue to slow the progression of β-cell failure. These findings support the potential of anthocyanin-rich dietary strategies not only for management but potentially for the prevention of T2D onset, offering a safe and accessible approach to safeguard metabolic health.

## 5. Conclusions

In conclusion, this systematic review demonstrates that anthocyanins consistently protect β-cells from diverse forms of metabolic stress through interconnected mechanisms involving antioxidant defence, anti-apoptotic signalling, and preservation of insulin secretory mechanisms. Although the *in vitro* nature of these findings limits direct clinical translation, the mechanistic consistency across models and stressors, combined with effects at physiologically relevant concentrations, suggests genuine therapeutic potential. These findings provide a mechanistic foundation for the observed benefits of anthocyanin-rich foods in clinical and epidemiological studies, supporting their role in dietary strategies for T2D prevention and management.

## Figures and Tables

**Table 1:** Summary of inclusion and exclusion criteria applied in the study selection process.

Parameters	Inclusion	Exclusion
Type of publication	Original articles	Reviews, letters, book chapters, conference abstracts
Year of publication	Until April 2025	
Kind of study	*In vitro* studies on antidiabetic activity of anthocyanins	in vivo, ex vivo, and clinical studies
Cell type	Primary islets (human and animals), β-cell lines	Other cell types and co-culture models
Compound	Glycosylated anthocyanins, purified or in extracts	Anthocyanidins, proanthocyanidin, procyanidins, acylated anthocyanins, leucocyanidins, or other bioactives
Measurements	At least one of the following: viability, apoptosis, glucose-stimulated insulin secretion, gene expression, or antioxidant activity	Studies lacking any of these measurements
Language	English	Language other than English

**Table 2:** Summary of *in vitro* studies investigating the effects of anthocyanins (ACNs) on β-cell function, viability, and stress responses.

Reference	Model	ACN (Source)	Stressor	Outcomes Measured	Dose	Time (h)	Main Findings
Cai et al., 2015, [38]	Mouse islets	C3G (Chinese bayberry)	None	Viability; Gene expression	0.1–5 µM	24	↑ Viability (max at 1, 5 µM); Gene: ↔ *Ho-1*, *Bcl-2*, and *Survivin*
Pilailak Channuwong et al., 2025, [30]	INS-1	M3G (commercial)	None	Viability; GSIS; Gene expression	1–300 µM	2–24	↑ GSIS (at 60, 100 µM); ↔ Viability (upto 100 µM); Gene: ↑ *Ins1*, *Scla2a2*, *Gck*, *Cacnac1c*, *Kcnj11* (max at 24 h)
Chen et al., 2022, [26]	INS-1 Mouse islet	C3G (commercial)	HG, PA	Apoptosis; GSIS; Gene expression; Protein expression	12.5–50 µM	12, 24, 48	↑ GSIS (only at 50 µM, 24 h with PA); ↓ Apoptosis (at 50 uM); Gene: ↑ *Glut2*, *Ins1*, *Ins2*, *Slc2ac*, ↓ *Chop*, *Perk* (at 50 µM) Protein: ↑ GPR40, ↓ TLR4, GRP78, p-EIF2α, IL-1β, p-PERK, CHOP, ↔TNF-α;
Choi et al., 2018, [27]	INS-1	C3R (commercial)	HG	Viability; GSIS; Protein expression; Antioxidant activity	10–50 µM	48	↑ Viability (max at 50 µM); ↑ BIS and GSIS (max at 50 uM; Protein: ↓ Cyto c, Cas 9, Cas 3, ↑ BCL-2/BAX; AA: ↑ SOD, CAT, GSH-px; AA: ROS, NO, TBARS ↓ (max at 50 µM)
Croden et al., 2021, [39]	Human islets	C3G (Chinese bayberry)	Amylin Aβ1-42 H_2_O_2_	Viability; GSIS; Gene expression; Antioxidant activity	1 µM	24	↑ Viability (1 µM), ↓ amyloid formation, ↑ GSIS; Gene: ↑ *HO*-*1*, *IL-1β*, ↑ *LC3* (Amylin), ↓ *LC3* (Aβ1-42 and H_2_O_2_), ↓ *NLRP3*; AA: ↓ ROS
Jeon et al., 2018, [40]	RIN-m5F	ARE (Aronia berry)	Cytokines	Viability; Gene expression; Antioxidant activity	1–1000 µg/mL	24	↔ Viability; Gene: ↓ *Cox-2* and *iNOS*; Protein: p-ERK, p-JNK, p-p38, p-NF-κB (max at 10–100 mg/mL); AA: ↓ NO (max at 1 mg/mL)
Johnson & de Mejia, 2016, [25]	INS-1	ARE (Blueberry Blackberry)	HG	GSIS; Gene expression; Protein expression	50–100 CE	0.5	↑ GSIS; ↑ 20+ genes; Protein: ↑ GLP-1R (at 100 CE)
Phutthida Kongthitilerd et al., 2022, [33]	INS-1	C3R (Synthesised from Rutin)	None	Viability; GSIS; Gene expression	3–300 µM	6, 24	↑ Viability (max at 100 µM); ↑ GSIS (max at 100 µM); Gene: ↑ *Glut2*, *Kir6.2*; ↔ *Cav1.2*, *GK* (100 µM)
LEE et al., 2015, [29]	MIN6	C3G (Mulberry)	HG	Viability; BIS; Apoptosis; Protein expression; Antioxidant activity	23–445 µM	18	↑ Viability (max at 156 µM); ↑ BIS (max at 156 µM); ↓ Apoptosis (max at 156 µM); Protein: ↑ Bcl2, ↓ Bax, Cyt c, ↓ NF-κB, ↓ ERK, JNK, p38; AA: ↓ ROS (max at 445 µM);
LEE, KIM, SONG, et al., 2015, [32]	MIN6N	C3G (Mulberry)	H_2_O_2_	Viability; BIS; Apoptosis; Antioxidant activity	23–1113 µM	20	↔ Viability (≤445 µM); ↑ BIS (max at 156 µM) ↓ Apoptosis (max at 156 µM); AA: ↓ ROS, LPO (max at 445 µM)
Luna-Vital et al., 2019, [41]	INS-1	ARE (Red maize)	None	GSIS; Protein expression	100–1000 µg/mL	2	↑ GSIS in INS-1 (max at 1mg/mL); Protein: ↑ PLC, FFAR1, p-PKD (max at 0.5 mg/mL)
Sun et al., 2012, [28]	INS-1	ARE (Chinese bayberry)	H_2_O_2_	Viability; Gene expression; Protein expression; Antioxidant activity	0.5 µM	24	↑ Viability; Gene: ↑ *Pdx-1*, *Ins2*; Protein: ↑ Insulin protein; AA: ↓ ROS
Ye et al., 2021, [42]	NIT	C3G (Chinese bayberry)	HG, PA	Viability; Protein expression; Antioxidant activity	22–445 µM	24	↑ Viability (max at 22 µM); Protein: ↑ PINK1, PARKIN, LC3; AA: ↓ ROS, ↑ SOD, CAT (max at 178 µM);
Zhang et al., 2013, [34]	INS-1	ARE (Chinese bayberry)	No	Protein expression	0.5–1 CE	24	Protein: ↓ LC3, ↑ HO-1
Zheng et al., 2016, [43]	Min 6	C3G, C3R (Mulberry)	H_2_O_2_	Viability; GSIS; Apoptosis; Protein expression; Antioxidant activity	12.5–50 µM	24	↑ Viability; ↑ BIS and GSIS; ↓ Apoptosis; Protein: ↑ GK, GLP-1R, PDX-1 (max at 50 µM); AA: ↓ ROS (12.5–50 µM)
Zhang et al., 2011, [44]	INS-1 Mouse islet	C3G (Chinese bayberry)	H_2_O_2_	Viability; Apoptosis; Gene expression; Protein expression	0.1–5 µM	12, 24, 48	↑ Viability; ↓ Apoptosis; Gene: ↑ Ho-1; Protein: ↓ Cas-3, -9; ROS ↓ (max at 1 µM);
Liu et al., 2015, [45]	INS-1 832/13	ARE (Chinese bayberry)	HG, PA	Viability; Apoptosis; GSIS; Antioxidant activity	1–100 µg/mL	24, 36	↑ Viability; ↓ BIS, ↑ GSIS; ↓ Apoptosis; AA: ↓ ROS, MDA; ↑ SOD (max at 10–100 µg/mL), ↑ GSH-Px (max at 1 µg/mL)
Jayaprakasam et al., 2005, [31]	INS-1 832/13	D3G, C3G, C3Gal, P3G; (Cornelian cherry)	HG	BIS	11–555 CE	24	↑ by D3G, C3G and C3Gal, but ↔ P3Gal

ACN = anthocyanin; ARE = anthocyanin-rich extract; C3G = cyanidin-3-glucoside; C3R = cyanidin-3-rutinoside; M3G = malvidin-3-glucoside; D3G = delphinidin-3-glucoside; C3Gal = cyanidin-3-galactoside; P3G = pelargonidin-3-glucoside; CE = C3G equivalents; HG = high glucose (glucotoxic stress); PA = palmitic acid (lipotoxic stress); BIS = basal insulin secretion; GSIS = glucose-stimulated insulin secretion; AA = antioxidant activity; ROS = reactive oxygen species; NO = nitric oxide; TBARS = thiobarbituric acid-reactive substances; MDA = malondialdehyde; SOD = superoxide dismutase; CAT = catalase; GSH-Px/GPx = glutathione peroxidase; LPO = lipid peroxidation; GK/Gck = glucokinase; GLUT2/Slc2a2 = glucose transporter-2; Kir6.2/Kcnj11 = ATP-sensitive K+ channel subunit; Cav1.2/Cacna1c = L-type voltage-gated calcium channel; PDX-1 = pancreatic and duodenal homeobox-1; GLP-1R = glucagon-like peptide-1 receptor; IGF-1R = insulin-like growth factor 1 receptor; FFAR1/GPR40 = free fatty acid receptor 1; PLC = phospholipase C; p-PKD = phosphorylated protein kinase D TLR4 = toll-like receptor 4; CHOP = C/EBP homologous protein; GRP78 = glucose-regulated protein 78; PERK = protein kinase R-like endoplasmic reticulum kinase; eIF2α = eukaryotic initiation factor 2 alpha; LC3 = microtubule-associated protein 1A/1B-light chain 3; PINK1 = PTEN-induced kinase 1; HO-1 = heme oxygenase-1; Nrf2 = nuclear factor erythroid 2–related factor 2; IL-1β = interleukin-1 beta; TNF- α = tumor necrosis factor alpha; NF-κB = nuclear factor kappa-light-chain-enhancer of activated B cells; COX-2 = cyclooxygenase-2; iNOS = inducible nitric oxide synthase; MAPK = mitogen-activated protein kinase; ERK = extracellular signal-regulated kinase; JNK = c-Jun N-terminal kinase; Cas3 = caspase-3; Cas9 = caspase-9; BCL-2 = B-cell lymphoma 2; BAX = BCL2-associated X protein; Cyt c = cytochrome c; Aβ_1_–_42_ = amyloid-β_1_–_42_; ↔ = no change; ↑ = significant increase/upregulation; ↓ = significant decrease/downregulation.

**Table 3:** Quality assessment of included *in vitro* studies using the modified ToxRTool framework.

Quality Assessment—Modified ToxRTool for *In Vitro* Studies		Score (0–2) Each Criterion																	
Domain	Assessment Criterion	1	2	3	4	5	6	7	8	9	10	11	12	13	14	15	16	17	18
Test Compound Characterization	Type and purity of anthocyanin defined (pure vs. extract; specific compound name)	2	2	2	2	2	2	2	2	2	2	2	2	2	2	2	2	2	2
	Source and preparation of anthocyanin or extract described (plant origin, supplier, extraction method)	2	1	1	1	2	2	2	1	2	2	2	2	2	2	2	2	2	2
	Concentration range and dosing method clearly reported (units and rationale)	1	1	1	1	1	1	1	1	1	1	1	1	1	1	1	1	1	1
Experimental Model Details	Cell line type and source stated (passage number, supplier)	2	2	1	1	2	1	1	1	1	1	1	2	1	0.5	1	0.5	1	1
	Exposure conditions specified (duration, medium, glucose concentration, vehicle)	2	2	2	2	2	2	2	2	2	2	2	2	2	2	2	2	2	2
Study Design Quality	Use of appropriate controls (vehicle, untreated, or positive control described)	2	2	2	2	2	2	2	2	2	2	2	2	2	0	2	2	2	1
	Replicates clearly stated (technical and/or biological replicates)	2	2	2	2	2	2	2	2	2	2	2	2	2	0	2	2	0	1
	Statistical analysis described and appropriate	2	2	2	2	2	2	2	2	2	2	2	2	2	2	2	2	2	2
Data and Reporting Transparency	Results presented with variability measures (mean ± SD, SE)	2	2	2	2	2	2	2	2	2	2	2	2	2	2	2	2	2	2
	Key outcome measures relevant to β-cell function reported (viability, apoptosis, insulin secretion, oxidative stress)	2	2	2	2	2	2	2	2	2	2	2	2	2	1	2	2	2	2
Mechanistic Insight	Mechanistic pathway analysis reported	0	2	2	2	1	1	2	2	2	2	2	1	2	1	2	1	0	0
Transparency	Disclosure of limitations or conflicts of interest	1	1	2	1	1	1	1	1	0	0	1	1	2	1	1	0.5	0.5	0.5
	Total score	20	21	21	20	21	20	21	20	20	20	21	21	22	14.5	21	19	16.5	16.5
	Quality category (High [H], Medium [M], Low [L])	H	H	H	H	H	H	H	H	H	H	H	H	H	M	H	H	H	H

Colour coding: green = adequately reported (score 2); orange = partially reported (score 1); red = not reported (score 0).

## Data Availability

No new data were created or analyzed in this study. Data sharing is not applicable to this article.

## Abbreviations

The following abbreviations are used in this manuscript:

ACN	anthocyanin
ARE	anthocyanin-rich extract
GSIS	glucose-stimulated insulin secretion
ROS	reactive oxygen species
NO	nitric oxide
HO-1	heme oxygenase-1
GLUT2	glucose transporter 2
C3G	cyanidin-3-glucoside
C3R	cyanidin-3-rutinoside
M3G	malvidin-3-glucoside
PDX-1	pancreatic and duodenal homeobox 1
Bcl-1	B-cell lymphoma 2
LC3	microtubule-associated protein 1 light chain 3
GPR40	G-protein-coupled receptor 40
SOD	superoxide dismutase
CAT	catalase
GSH-Px	glutathione peroxidase
IL-1β	interleukin-1 beta
TNF	α-tumor necrosis factor alpha
